# Gastrectomy alters emotional reactivity in rats: neurobiological mechanisms

**DOI:** 10.1111/j.1460-9568.2011.07640.x

**Published:** 2011-05

**Authors:** Nicolas Salomé, Magdalena Taube, Emil Egecioglu, Caroline Hansson, Björn Stenström, Duan Chen, Daniel R Andersson, H Georg Kuhn, Claes Ohlsson, Suzanne L Dickson

**Affiliations:** 1 Department of Physiology/Endocrinology, Institute of Neuroscience and Physiology, The Sahlgrenska Academy at the University of GothenburgMedicinaregatan 11, SE-405 30 Gothenburg, Sweden; 2 Department of Cancer Research and Molecular Medicine, Norwegian University of Science and TechnologyTrondheim, Norway; 3 Department of Pharmacology, Institute of Neuroscience and Physiology, The Sahlgrenska Academy at the University of GothenburgGothenburg, Sweden; 4 Center for Brain Repair and Rehabilitation, Institute of Neuroscience and Physiology, The Sahlgrenska Academy at the University of GothenburgGothenburg, Sweden; 5 Research Center for Endocrinology and Metabolism, The Sahlgrenska Academy at the University of GothenburgGothenburg, Sweden

**Keywords:** anxiety, depression, gastric surgery, gut hormones

## Abstract

Gastrectomy (Gsx) is associated with altered emotional function and a predisposition to depression/anxiety disorders. Here we investigated the effects of Gsx on emotional reactivity in rats and explored the underlying neurobiological mechanisms. Gsx- and sham-operated rats were exposed to behavioural tests that explore anxiety- and depression-like behaviour (open field, black and white box, elevated plus maze, social interaction, forced swim) as well as memory (object recognition). The potential neurobiological mechanisms underlying these differences were explored by measuring (i) turnover of candidate neurotransmitter systems in the nucleus accumbens, (ii) hippocampal neurogenesis by BrdU labelling or by analysis of candidate genes involved in neuronal growth and (iii) changes in mRNA expression of candidate genes in dissected hippocampal and amygdala tissue. Data from individual behavioural tests as well as from multivariate analysis revealed differing emotional reactivity between Gsx- and sham-operated rats. Gsx rats showed reduced emotional reactivity in a new environment and decreased depression-like behaviour. Accumbal serotonin and dopamine turnover were both reduced in Gsx rats. Gsx also led to a memory deficit, although hippocampal neurogenesis was unaffected. Of the many candidate genes studied by real-time RT-PCR, we highlight a Gsx-associated decrease in expression of Egr-1, a transcription factor linked to neural plasticity and cognition, in the hippocampus and amygdala. Thus, Gsx induces an alteration of emotional reactivity and a memory/cognitive deficit that is associated with reduced turnover of serotonin and dopamine in the nucleus accumbens and decreased expression of Egr-1 in the hippocampus and amygdala.

## Introduction

As recently as 1999, gastric carcinoma was reported to be the second largest cause of cancer death in the world (Liedman, 1999), although its incidence has been continuously decreasing during the past decade. Total gastrectomy (Gsx) is still a mainstay treatment for this cancer and is needed in approximately 60% of cases. Following surgery, a variety of symptoms and impairments emerge, collectively referred to as the ‘postgastrectomy syndrome’ (Liedman, 1999). These include reduced appetite, weight loss, osteopaenia and malnutrition. Loss of reservoir function of the stomach, dysphagia, dyspepsia, altered intestinal motility, malabsorption and so-called ‘gastric dumping syndrome’ have been offered as explanations for the weight loss.

Gsx disrupts the endocrine milieu, including that of circulating hormones regulating food intake and energy balance such as the orexigenic and pro-obesity hormone ghrelin (Ariyasu *et al.*, 2001; Dornonville de la Cour *et al.*, 2001) and also the anorexigenic hormones cholecystokinin (CCK; Inoue *et al.*, 1987) and leptin (Furnes *et al.*, 2008a). We have demonstrated beneficial effects of ghrelin replacement for Gsx-associated suppression of body weight and body fat (Dornonville de la Cour *et al.*, 2005) reflecting direct hypothalamic effects on genes regulating energy balance (Egecioglu *et al.*, 2008). We were unable to explain Gsx-induced weight loss by suppressed food intake or increased energy expenditure (Dornonville de la Cour *et al.*, 2005) although in a subsequent study in rats, we did observe a reduction in cumulative food intake (Furnes *et al.*, 2008a).

In addition to metabolic disturbance, patients who have undergone Gsx for gastric cancer appear to be predisposed to depression/anxiety disorders and have an altered emotional function (Everitt *et al.*, 1995; Wu *et al.*, 2000; Fujita *et al.*, 2003; Hjermstad *et al.*, 2006). Given the emerging neurobiology concerning the role of gut hormones in psychiatric diseases such as anxiety disorders and depression (Asakawa *et al.*, 2001; Hernandez-Gomez *et al.*, 2002; Kanehisa *et al.*, 2006; Lutter *et al.*, 2008) as well as memory (Hebb *et al.*, 2005; McNay, 2007), together with the neurochemical overlap between metabolic and psychiatric disease, it seems likely that the endocrine stomach has a role in the emotionality changes that accompany Gsx. Here we sought to determine whether Gsx rats differ from sham-operated rats in terms of (i) their behavioural responses when subjected to tests frequently used to evaluate anxiety, depression and memory in rodents, (ii) their brain neurochemistry (turnover of transmitter systems involved in emotional reactivity in relevant brain regions), (iii) nerve cell regeneration (hippocampus) and (iv) the expression patterns of genes involved in anxiety/depression, nerve cell regeneration, plasticity and signal transduction in brain areas implicated in the neuropathology of affective disorders (Campbell & Macqueen, 2004; Mathew *et al.*, 2008).

## Methods

### Animals and experimental procedures

Gsx or sham operation was performed on age-matched adult male Sprague–Dawley rats (weighing approximately 155 g at the start of the experiment, obtained from B&K Universal, Sollentuna, Sweden). They were group-housed upon arrival and maintained in a 20 °C, 40–45% humidity and 12-h light/dark cycle with free access to standard rat chow (Harlan Teklad, Norfolk, UK) and tap water. All surgical procedures were performed in anaesthetized animals (60–75 mg/kg Ketalar® and 0.5 mg/kg Domitor® i.p.; Pfizer, Sweden; Orion Co., Finland) through a short upper midline incision. Sham operation consisted of laparotomy. Gsx was performed by removing the whole stomach followed by joining the oesophagus and duodenum end-to-end. Animals were allowed to recover from surgery for 3 weeks before commencing the behavioural testing. All animal experiments were approved by the local Ethics Committee in Gothenburg, Sweden. Twenty rats were used in the study: 10 sham rats and 10 Gsx rats. Four of the Gsx rats experienced post-operative complications resulting in excessive weight loss and were killed prior to the behavioural testing according to local Ethics Committee guidelines.

### Behavioural testing for emotional reactivity and memory testing

Emotional reactivity was explored in behavioural tests designed to explore anxiety-like behaviour (the open field test, the black and white box and the elevated plus maze; Ramos & Mormede, 1998; Cryan *et al.*, 2002) and depression-like behaviour (the forced swim test; Porsolt *et al.*, 1977). The object recognition test was used to assess memory performance (Carlini *et al.*, 2008).

To facilitate adaptation, Gsx and sham animals were placed in an ante-room 1 h prior to all behavioural testing. The tests were carried out during the light phase, between 08:30 and 11:30 h, using a balanced design and in the order as listed below. Behaviour was recorded via a camera directly linked to a computer located in an adjacent room. All of the behavioural analyses were done by an observer who was unaware of the individual surgical manipulation.

#### Open field test

The open field consisted of a wood box (60 × 60 × 60 cm^3^). The illumination at floor level was 700 lux. The open field was divided into a 25 × 25-cm central zone and a border zone surrounding it. To evaluate locomotor activity, the floor was divided into 16 equal squares. At the beginning of the test, rats (sham, *n*=10; Gsx, *n*=6) were individually placed in the open field and their behaviour was analysed during the 10-min test period: time spent in the central zone, the number of entries into the central zone, the number of groomings and the time spent grooming, the number of rearings in the central and peripheral zone and the number of line crossings. The field was cleaned between each test session.

#### Object recognition

The day after the open field test, object recognition memory was assessed using the object recognition task (sham, *n*=9; Gsx, *n*=5). Trials consisted of a sample trial (T1) and a recognition trial (T2). The two trials were separated by an inter-trial interval of 24 h. During T1, rats were placed in the enclosure in the presence of two identical objects for 3 min. Any animal not exploring (defined as the animal having its head within 2 cm of the object while looking at, sniffing or touching it) the objects for 20 s within the 3-min period was not included in the experiment.

During T2, the rats were again placed in the enclosure with a previously presented familiar object together with a novel object for a period of 3 min. Time spent exploring the familiar and novel objects was recorded. The percentage of time spent exploring the new object was calculated. Animals exploring the objects for < 10 s were removed from the study. The objects used were a white wood cylinder and a green wood pyramid. Two different sets of objects were used to enable them to be wiped between one rat and the next, to prevent the possibility of olfactory recognition.

#### Black and white box

The box was made of wood and divided in two compartments, connected by an opening (5 × 5 cm^2^). The first compartment (18 × 27 × 27 cm^3^) was painted black and covered by a black top giving an illumination around 10 lux. The other compartment (27 × 27 × 27 cm^3^) was painted white and was lit by a white incandescent bulb (100 lux). The floor of the box was cleaned before each trial. At the beginning of each 10 min trial, the rat (sham, *n*=10; Gsx, *n*=6) was placed in the centre of the white compartment facing the opening. The behavioural parameters scored were: the latency until the first entry into the black compartment, the time spent in the white compartment, the number of transitions between the black and the white compartment, the number of rearings in both compartments, the number of groomings and the time spent grooming.

#### Elevated-plus maze

The apparatus consisted of two open arms (50 × 10 cm^2^) surrounded by a 1-cm-high plexiglass and two closed arms (50 × 10 × 38 cm^3^) that emerge from a central platform. The apparatus was made from dark grey PVC and the arms were elevated 73 cm above the floor. A white incandescent bulb provided a light intensity of 100 lux over the open arms and of 20 lux over the closed arms. At the beginning of each trial, the rat (sham, *n*=10; Gsx, *n*=6) was placed on the central platform facing a closed arm. The floor of the maze was cleaned before each trial. The rat behaviour was videotaped over 5 min and a trained observer scored the parameters from the videotape. The behavioural parameters scored were: the number of entries into the closed arms and in all arms, the number of open arm entries (expressed as a percentage of the total number of entries; an entry was counted when both forepaws were placed on the respective arm), the time spent there (expressed as a percentage of the total time spent in all arms), the total time spent on all arms and the percentage of time spent in the central platform.

#### Forced-swim test

The procedure was a modification of that described by Porsolt *et al.* (1977). Animals (sham, *n*=10; Gsx, *n*=6) were placed in an individual glass cylinder (diameter 17 cm, height 40 cm) containing water (height 24 cm, 22 °C). Two sessions were conducted (an initial 15-min pre-test and, on the next day, a 5-min test). The duration of immobility, struggling and swimming (in seconds) were manually measured during each 5-min period of the pre-test sessions and during the 5 min of the test sessions by an experimenter who was unaware of the surgical groups. Immobility was defined as the minimal movement necessary for the rat to stay afloat. Floating occurred when rats remained immobile with only occasional slight movements to keep the body balance and the nose above the water. Immobility was assigned when no additional activity was observed other than that necessary to keep the rat's head above the water. Swimming occurred when rats moved all four limbs, swimming around in the tank or diving. Struggling was recorded when rats moved strongly all four limbs with the front paws breaking the water surface or scratching the glass cylinder wall.

### Dissection, blood collection and corticosterone measurement

Eight weeks following surgery and 2 weeks following the last behavioural experiment, body weight was recorded and the rats were killed by decapitation. Blood was collected from the trunk into EDTA-containing tubes immediately after decapitation and plasma was prepared. The adrenal glands and the thymus were dissected and weighed, as such organs typically have reduced weight in chronic stress paradigms that lead to depression. Brains were removed and dissected regions (hippocampus, amygdala, nucleus accumbens and prefrontal cortex) were frozen in liquid nitrogen and stored at −80 °C. Plasma corticosterone, also an indicator of chronic stress, was measured in plasma using a radioimmunoassay kit (MP Biomedical, Orangeburg, NY, USA) with ^125^I-corticosterone as standard.

### Content of monoamines and metabolites

The nucleus accumbens and prefrontal cortex were homogenized using a Sonifier B30 (Branson Sonic Power Co., Danbury, CT, USA) in 0.1 m HClO_4_ containing Na_2_EDTA (2.5 mm). After centrifugation (10 000 ***g***, 4 °C, 10 min), the supernatant was immediately analysed for dopamine (DA), serotonin (5-HT), dihydroxyphenylacetic acid (DOPAC), 5 hydroxyindolacetic acid (5-HIAA) and homovanillic acid (HVA) using a split fraction HPLC – electrochemical detection system. DA and 5-HT were separated on an ion-exchange column (Nucleosil, 5 μ SA 100 A, 150 × 2 mm; Phenomenex, Torrance, CA, USA) with a mobile phase consisting of 0.049 m citric acid, 0.0114 m NaOH and 0.012 mm Na_2_ EDTA. Ten sham rats and six Gsx rats were evaluated.

### Quantification of candidate genes in hippocampus and amygdala

Total RNA was individually prepared from the hippocampus and amygdala using an RNeasy Mini Lipid tissue kit (Qiagen, Hilden, Germany) with additional DNAse treatment (Qiagen). Isolated RNA was diluted in nuclease-free water (Ambion), and RNA concentrations were determined using a spectrophotometer. RNA integrity was ensured using the RNA 6000 Nano kit and an Agilent 2100 Bioanalyzer (Agilent Technologies).

For cDNA synthesis, total RNA corresponding to 1 μg was reversed transcribed in a total volume of 20 μL, using random hexamers (Applied Biosystems, Sundbyberg, Sweden) and Superscript III reverse transcriptase (Invitrogen Life Technologies, Paisley, UK), according to the manufacturer's description. Recombinant RNaseout® Recombinant Ribonuclease Inhibitor (Invitrogen) was added to prevent RNase-mediated degradation. All the cDNA reactions were run in triplicate.

TaqMan® Custom Array platforms were designed with TaqMan probe and primer sets for target genes chosen from an on-line catalogue (Applied Biosystems). The sets were factory-loaded into the 384 wells of TaqMan® Arrays. Reactions consisting of 2 μL cDNA (to a final concentration of 100 ng starting RNA) combined with 48 μL nuclease-free water and 50 μL TaqMan® Gene Expression Master Mix (Applied Biosystems) were loaded to each port. Duplicates of cDNA were run on separate LDA cards and analysed using the 7900HT system with a TaqMan LDA Upgrade (Applied Biosystems). Thermal cycling conditions were: 50 °C for 2 min, 94.5 °C for 10 min, 97 °C for 30 s and 59.7 °C for 1 min.

To calculate the expression stability of five reference genes [ribosomal protein L27, 18S rRNA, β-actin, glyceraldehyde-3-phosphate dehydrogenase (GAPDH) and Cyclophilin A] the NormFinder algorithm (http://www.mdl.dk/publicationsnormfinder.htm) was used. Gene expression values were calculated based on the ΔΔ*C*_t_ method (Livak & Schmittgen, 2001), where the saline-treated group was designated the calibrator. Briefly, Δ*C*_t_ represents the threshold cycle (*C*_t_) of the target minus that of the reference gene and ΔΔ*C*_t_ represents the Δ*C*_t_ of each target minus that of the calibrator. Relative quantities (RQ) or fold changes were determined using the equation: relative quantity = 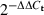. For the calibrator sample, the equation is relative quantity = 2^−0^, which is 1; therefore, every other sample is expressed relative to this.

Target genes included transcription and neural growth factors as well as genes involved in receptor and transporter activity. All TaqMan assay identities are listed in Table 1.

**Table 1:** Gene targets and TaqMan assay identities (id) loaded on the Taq®Man Array

Gene target	Gene name	Assay id
Endogenous controls		
18S	Eukaryotic 18S rRNA	Hs99999901_s1
Actb	Actin, beta	Rn00667869_m1
Gapdh	Glyceraldehyde-3-phosphate dehydrogenase	Rn99999916_s1
Ppia	Peptidylprolyl isomerase A	Rn00690933_m1
Rplp2	Ribosomal protein, large P27	Rn01479927_g1
Transcriptor factor/signal transduction		
Crebbp	CREB binding protein	Rn00591291_m1
Egr1	Early growth response 1	Rn00561138_m1
Map2k2	Mitogen activated protein kinase kinase 2	Rn00590971_m1
Trh	Thyrotropin-releasing hormone	Rn00564880_m1
Transporter/enzymatic activity		
Gad1	Glutamic acid decarboxylase 1 (67)	Rn00566593_m1
Th	Tyrosine hydroxylase	Rn00562500_m1
Slc1a2	Glt1 solute carrier family 1 (glial high affinity glutamate transporter), member 2	Rn00568080_m1
Slc6a3	Dat1 solute carrier family 6 (neurotransmitter transporter, dopamine), member 3	Rn00562224_m1
Slc6a4	5HTT neurotransmitter transporter, serotonin	Rn00564737_m1
Neural growth/differentiation		
Bdnf	Brain-derived neurotrophic factor	Rn01484928_m1
Ntrk2	Neurotrophic tyrosine kinase, receptor, type 2	Rn00820626_m1
Ncam1	Neural cell adhesion molecule 1	Rn00580526_m1
Ngfb	Nerve growth factor beta	Rn01533872_m1
Receptor activity		
Adipor1	Adiponectin receptor 1	Rn01114954_g1
Cckbr	Cannabinoid receptor1	Rn00565867_m1
Drd3	Dopamine receptor 3	Rn00567568_m1
Gabra1	Gamma-aminobutyric acid A receptor, alpha1	Rn00788315_m1
Gabra3	Gamma-aminobutyric acid A receptor, alpha3	Rn00567055_m1
Gabra4	Gamma-aminobutyric acid A receptor, alpha4	Rn00589846_m1
Gabra5	Gamma-aminobutyric acid A receptor, alpha5	Rn00568803_m1
Galr2	Galanin receptor 2	Rn01773918_m1
Galr3	Galanin receptor 3	Rn02132531_s1
Ghsr	Growth hormone secretagogue receptor	Rn00821417_m1
Htr1a	5-hydroxytryptamine (serotonin) receptor 1A	Rn00561409_s1
Htr2c	5-hydroxytryptamine (serotonin) receptor 2C	Rn00562748_m1
Insr	Insulin receptor	Rn00567070_m1
Lepr	Leptin receptor	Rn01433205_m1

### Hippocampal neurogenesis

Treatment with BrdU through once daily intraperitoneal injections started 3 days following the surgical procedure and continued for 17 consecutive days. BrdU (Sigma, St. Louis, MO, USA) was dissolved in 0.9% NaCl at a concentration of 10 mg/mL and given at 50 μg/kg/day. Eight weeks following surgery animals were perfused transcardially with 4% paraformaldehyde in phosphate buffer. Brains were removed, stored in fixative overnight, transferred into 30% sucrose and frozen at −80 °C. The left hemisphere was cut in sagittal sections at 40 μm on a sliding microtome (Leica SM2000R; Leica Microsystems, Nussloch, Germany). Serial sections were collected and stored in tissue cryoprotection solution (TCS: 25% glycerine, 25% ethylene glycol and 50% 0.1 n phosphate buffer) until subsequent processing for immunocytochemistry.

From each animal, every 12th section was selected for immunofluorescence staining for BrdU, Neu-N and Prox-1. The sections were rinsed using Tris-buffered saline (TBS) for 3 × 10 min, pre-treated in 2 m HCl at 37 °C for 30 min to denaturate DNA and rinsed in 0.1 m borate buffer (pH 8.5) for 10 min at room temperature. After pre-treatment and blocking in 3% normal donkey serum (Jackson ImmunoReasearch Laboratories Inc., Suffolk, UK) and 0.1% Triton X100 in TBS for 30 min, sections were incubated in an antibody mixture containing antibodies against BrdU (1 : 250 Rt anti-BrdU; Nordic Biosite AB, Sweden), NeuN (1 : 100 Ms anti-NeuN; Chemicon International, USA) and Prox-1 (1 : 1000 Rb anti-Prox1; Chemicon) at 5 °C overnight. Following incubation with primary antibody the sections were rinsed with TBS and incubated in the dark for 2 h at room temperature with secondary antibodies (Dk anti-Rt IgG 488 for BrdU detection, Dk anti-Ms IgG 647 for NeuN detection and Dk anti-Rb IgG 555 for detection of Prox1; Molecular Probes Inc., USA), all at dilutions of 1 : 1000. Sections were mounted onto Super Frost Microscope slides, coverslipped with ProLong Gold antifade reagent containing DAPI (4′,6-diamidino-2-phenylindole) and finally stored in the dark to prevent fluorescence fading.

Cell counting comprised counting the BrdU-labelled cells seen with the N3 filter cube in a stereo microscope (Leica DM 6000 B). Prox-1 staining in the green channel was used to better visualize the dentate gyrus. To determine the number of neural precursor cells that matured into neurons, 50 BrdU-positive cells per animal were randomly analysed for NeuN staining, giving a ratio of the amount of NeuN/BrdU-positive double-labelled cells. Phenotyping of Brdu/BrdU-NeuN double labelled cells was performed with a confocal microscope (Leica DM IRE2) by non-sequential scanning combined with z-scanning. The analysis was done on 50 cells per animal in which the BrdU was visualized with the 488-nm laser line and the NeuN using the 647-nm laser line.

### Statistical analysis

Data are expressed as means ± SEM. Data obtained were analysed either by a Student's *t*-test or by a Mann–Whitney *U*-tests when data were not normally distributed or were of unequal variance. The results of the forced swim test obtain during the second day were compared with those obtained on the first day using a two-way anova (time × surgery) followed by a HSD Tukey *post hoc* test for comparisons between groups when appropriate. In each case, *P*<0.05 was considered statistically significant.

A discriminant analysis was performed for each test (except for the object recognition) to test the accuracy of divergence in emotional reactivity between the two surgical groups. Different parameters given by this analysis were reported: a classification matrix was used to determine to which groups (sham vs. Gsx groups) each animal tested most likely belonged (results expressed as a percentage), the discriminating power of each variable was given by the *F*-values (indicating its statistical significance in the discrimination between groups), and the tolerance value was a measure of the redundancy for a variable with all previous variables included in the model. When a variable was highly redundant, its tolerance value and its contribution to the model were low.

In addition, to dissect the specific emotional dimensions in each surgical group, a principal components analysis was performed using parameters obtained in the different tests: the open field test (number of rearings in the periphery, number of line crossings, number of groomings), the black/white box (number of rearings in the white compartment, number of groomings), the elevated plus-maze (number of rearings in the closed arms, percentage of time spent in the open arms, percentage of time spent in the central platform) and the forced swim test (time swimming during the first triad, time spent struggling during the second triad, time spent swimming the second day). These variables have been chosen to avoid keeping variables with a correlation > 0.9. An orthogonal rotation (varimax) of the factor matrix was used to analyse the data, ensuring that the extracted factors were independent of each other. Considering the sample size, a Spearman correlation matrix, which is less sensitive for single outliers, was used (Gorsuch *et al.*, 1998). An orthogonal rotation (varimax) of the factor matrix was used to analyse the data, which ensured that the extracted factors were independent. Only factors with eigenvalues > 0.4 were kept for analysis.

## Results

### Body weight, organ weight and hormone measurements

Body weight before surgery was not different (sham group, 156 ± 1 g; Gsx group, 154 ± 2 g; *t*_12_ = 1.26, *P*=0.23). At 8 weeks after surgery (at the end of the experiment), the Gsx rats had a lower body weight than the sham group (294 ± 18 and 508 ± 14 g, respectively, *t*_12_ = 9.65, *P*=0.00001). In the Gsx group, absolute weights of the thymus and adrenal glands were reduced and tended to be reduced, respectively (thymus: sham group, 1.73 ± 0.05 mg; Gsx group, 1.4 ± 0.03 mg, *t*_10_ = 5.57, *P*=0.00024; adrenal: sham group, 0.961 ± 0.005 mg; Gsx group, 0.946 ± 0.004 mg, *t*_12_ = 2.91, *P*=0.012; data not shown). However, when adjusted for body weight, the weights of these organs were increased (thymus: sham group, 0.26 ± 0.01 mg; Gsx group, 0.37 ± 0.02 mg, *t*_10_ = −6.57, *P*=0.000063; adrenal: sham group, 0.19 ± 0.01 mg; Gsx group, 0.33 ± 0.02 mg, *t*_12_ = −7.93 *P =*0.000004; data not shown). Plasma corticosterone levels were reduced by Gsx (sham group, 393 ± 38 ng/mL; Gsx group, 116 ± 37 ng/mL, *t*_12_ = 5.59, *P*=0.00016).

### Behavioural studies of anxiety in sham-operated and Gsx rats

**Fig. 1:**
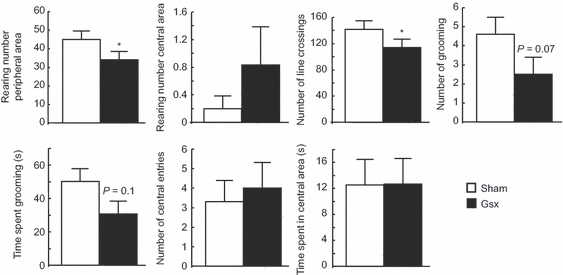
Behavioural parameters of sham-operated (*n*=10) and Gsx rats (*n*=6) in the open field during the 10-min test period. Columns and bars represent mean ± SEM. *P <0.05.

There are indications from individual behavioural tests of anxiety-like behaviour that emotional reactivity differs between sham-operated and Gsx rats. In the open field test, the number of peripheral rearings and the number of line crossings were lower in Gsx rats than in sham-operated rats (*t*_14_ = 2.67, *P =*0.018 and *t*_14_ = 2.938, *P*=0.01). The final number of surviving operated rats in the two experimental groups was relatively low for a behavioural study (i.e. six Gsx and ten sham-operated), yet it seems clear that the two experimental groups had an different overall emotional response (Fig. 1). No significant difference was observed regarding the time spent grooming (*U*=16, *P =*0.13), the number of central rearings (*U*=22.5, *P =*0.41), the number of entries in the central area (*t*_14_ = −0.63, *P =*0.6) and the time spent in the central area (*t*_14_ = −0.03, *P =*0.97). In this test, the number of groomings tended to be lower in the Gsx group although this did not reach statistical significance (*t*_14_ = 1.9, *P =*0.07). In the black and white box (Table 2) the number of groomings also tended to be lower in the Gsx group (*U*=14, *P =*0.08) but otherwise there was no significant difference between the Gsx and sham groups for any of the other parameters measured (time spent grooming, *U*=16, *P*=0.13; latency until the first entry into the black compartment, *t*_14_ = 0.01, *P*=0.99; time spent in the white compartment, *t*_14_ = 0.69, *P*=0.49; number of transitions, *t*_14_ = 1.09, *P*=0.29; number of rearings in the white and black compartment, *t*_14_ = 0.89, *P*=0.38 and *t*_14_ = 0.54, *P*=0.59, respectively). Similarly, in the elevated plus maze (Table 2) there was no difference between groups for any parameter measured (number of closed arm entries, *t*_14_ = −0.49, *P*=0.63; number of total arm entries, *t*_14_ = −0.12, *P*=0.9; percentage of open arm entries, *t*_14_ = 0.34, *P*=0.73; percentage of time spent in open arms, *t*_14_ = −1.56, *P*=0.14).

**Table 2:** Behavioural parameters of sham-operated (*n*=10) and Gsx rats (*n*=6) in the black and white box and elevated plus maze

	Sham	Gastrectomy
Black and white box		
Number of groomings	2.6 ± 0.3	1.7 ± 0.2
Time grooming (s)	31.0 ± 6.2	16.7 ± 1.9
Latency first white entry (s)	2.6 ± 1.7	2.5 ± 0.9
Time in white (s)	23.5 ± 7.9	15.9 ± 5.2
Transition number	3.2 ± 0.8	2.0 ± 0.4
Rearing number in white	2.9 ± 1.1	1.5 ± 0.8
Rearing number in black	14.7 ± 1.7	13.2 ± 2.3
Elevated plus maze		
Closed arm entries	4.9 ± 0.8	5.5 ± 0.8
Total arm entries	8.2 ± 1.0	6.0 ± 1.1
Percentage open arm entries	34.1 ± 4.5	24.3 ± 2.8
Percentage time spent open arm	10.1 ± 2.8	8.7 ± 2.8
Percentage time spent central platform	3.6 ± 1.3	7.0 ± 1.8

The test periods were 10 min in the black and white box and 5 min in the elevated plus maze. Values are expressed as mean ± SEM.

### Behavioural studies of depression in sham-operated and Gsx rats – forced swim test

**Fig. 2:**
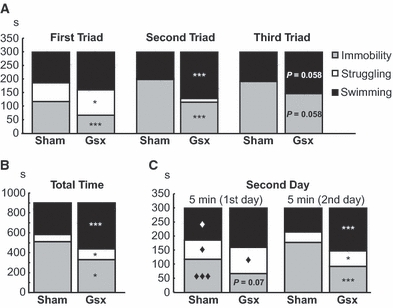
Behavioural parameters of sham-operated (*n*=10) and Gsx rats (*n*=6) in the forced swim test during (A) the three triads of the first day, (B) the 15 min of the first day and (C) the first 5 min of the 15 min pre-test session at the first day and during the test phase (5 min) on the second day. *P <0.05, ***P<0.001 Gsx vs. sham; ^♦^*P*<0.05, ^♦♦♦^*P <*0.001 when comparing day 1 and 2 for each surgical group.

In the forced swim test a decrease in immobility and an increase in active movements is indicative of a decrease in depression-like behaviour (Porsolt *et al.*, 1977). On the first day of the forced swim test, behavioural measurements were made in sequential 5-min triads (Fig. 2A and B) and on the second day they were made over one 5-min period only (Fig. 2C). On the first day, during the first triad, the Gsx group spent less time immobile (*U*=5, *P*=0.0066) and more time struggling (*t*_14_ = −2.22, *P*=0.43). During the second triad, time spent immobile was lower in the Gsx group, whereas the time spent swimming was increased (*t*_14_ = 3.46, *P*=0.0038 and *t*_14_ = −2.99, *P*=0.0095). In the third triad, the data were similar to the second triad but with only a tendency towards significance (*t*_14_ = 2.06, *P*=0.058 and *t*_14_ = −2.07, *P*=0.057). During the entire 15 min of exposure, Gsx rats displayed a decrease in the amount of time spent immobile (*t*_14_ = 3.44, *P*=0.0039) while they increased both the time spent swimming (*t*_14_ = −2.85, *P*=0.012) and the time struggling (*t*_14_ = −2.6, *P*=0.02, Fig. 2B).

anova revealed an interaction between day and surgical group (*F*_1,14_ = 8.05, *P <*0.05). On the second day, Gsx rats spent less time immobile and more time swimming in comparison with sham-operated rats (both *P <*0.001, Fig. 2C). When comparing the 2 days, the sham-operated rats spent more time immobile on the second day (*P <*0.001) and there was a similar trend for the Gsx rats (*P =*0.07). For both surgical groups the time spent struggling was lower on the second day than on the first day (*P <*0.05); however, for both days studied the Gsx rats tended to spend more time struggling compared with the sham-operated rats (*P <*0.05). Finally, anova revealed an interaction between day and surgery (*F*_1,14_ = 8.28, *P <*0.05). In addition, in comparison with the first day, sham-operated rats spent less time swimming on the second day (*P*<0.05) whereas Gsx rats did not modify their swimming behaviour between the first and the second day (*P =*0.67).

### Discriminant and multivariate analysis of the behavioural tests

A discriminant analysis was performed to test the accuracy of divergence between the two surgical groups (Table 3). The percentage correct discrimination as well as the discriminating factors between the groups are given, together with their corresponding *F* and *P* values and tolerance values. Gsx- and sham-operated and rats were markedly divergent with a high percentage of discrimination in all tests used. For the different anxiety tests, the most important parameters that discriminated Gsx- and sham-operated rats were: the number of line crossings in the open field test, the number of rearings in the white compartment in the black and white box, the percentage of time spent on the central platform and the percentage of open arm entries in the elevated plus maze. In the forced swim test, the most important distinguishing parameters were the time spent immobile during the first triad on the first day and the time spent swimming on the second day.

**Table 3:** Results from the discriminant analysis of sham-operated and Gsx rats in the four behavioural tests

	Percentage correct					
					
Test	Sham	Gsx	Discriminating variables	*F*	*P*	Tolerance
Open field	100	83.3	Line crossings	5.44	0.03	0.99
			Time grooming	0.66	0.23	0.99
Black and white box	80.0	83.3	Rearing white	4.39	0.05	0.58
			Grooming time	1.11	0.31	0.32
			Grooming latency	1.10	0.31	0.39
			Grooming number	0.42	0.42	0.39
Elevated plus maze	90.0	83.3	Percentage time centre	6.78	0.02	0.60
			Percentage entries open arm	4.95	0.04	0.60
Forced swim test						
1st day	90.0	100	Immobility (1st triad)	11.11	0.01	0.55
			Struggling (2nd triad)	2.57	0.13	0.58
			Struggling (3rd triad)	0.62	0.44	0.91
			Struggling (total)	0.02	0.87	0.57
2nd day	100	83.3	Swimming	24.78	0.00	0.99
			Struggling	1.21	0.29	0.99

For each test the percentage of correct classification of sham/Gsx animals is given. Within each test, variables were organized with the highest discriminating power first, as indicated by the *F*-value. The corresponding *P*-level was given for each *F*. The tolerance value is a measure of the redundancy of a variable with other variables in the same test (a higher tolerance indicates lower redundancy).

**Fig. 3:**
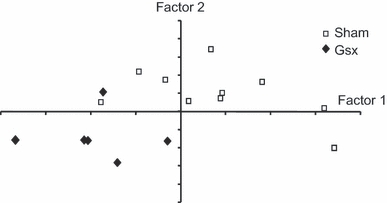
Distribution of sham-operated/Gsx rats along the two first factors extracted with the principal components analysis. Factor 1 was represented by the horizontal axis and accounts for 27.25% of the total variance. Factor 2 was represented by the vertical axis and accounts for 20.82% of the total variance. This principal components analysis was based on the behavioural results obtained in the open field (number of rearings at the periphery, number of line crossings, number of groomings), the black and white box (number of rearings in the white compartment, number of groomings), the elevated plus maze (number of rearings in the closed arms, percentage of entries into the open arms, percentage of time spent in the central zone) and in the forced swim test (immobility during the first triad, struggling during the second triad, time spent swimming the second day).

To determine the main factor(s) that discriminate sham-operated and Gsx rats, a principal components (multivariate) analysis based on the mean values of each group was performed for variables of the behavioural tests (Table 4). With the component analysis the 11 variables could be associated in four principal components with eigenvalues higher than 1 and representing 76.4% of the total variability. As the different factors were orthogonal to each other, it was generally assumed that they reflected distinct biological phenomena. To facilitate interpretation of the data, we present only the most significant loadings (higher than 0.4) in Table 4. The first factor represented 27.25% of the total variance. The parameters that correlated with this factor were: (i) in the open field, the number of rearings in the peripheral area and the number of line crossings; (ii) in the black and/white box, the number of rearings in the white compartment; and (iii) in the elevated plus maze, the percentage of open arm entries. The second factor represented 20.82% of the total variance. The parameters that loaded highly on this factor were: (i) in the black and white box, the number of groomings; (ii) in the elevated plus maze, the percentage of time spent on the central platform; and (iii) in the forced swim test, the time spent immobile during the first triad on the first day and the time spent swimming on the second day. The third factor represented 16.29% of the total variance. The parameters loading with this factor was derived mainly from the black and white box, i.e. the number of rearings in the white compartment and the number of groomings, but also the number of rearings in the closed arm of the elevated plus maze. The last factor represented 12.23% of the total variance. The parameters that correlated with this factor were: (i) in the open field, the number of line crossings and the number of groomings; (ii) in the elevated plus maze, the percentage of time spent on the central platform; and (iii) in the forced swim test, the time spent struggling during the second triad of the first day. The position of each individual animal of the two groups in relation to the first and the second factors is illustrated in Fig. 3.

**Table 4:** Principal components (multivariate) analysis of 11 variables obtained from the open field, black and white box, elevated plus maze and forced swim tests

Test/variable	Factor 1	Factor 2	Factor 3	Factor 4
Open field				
Rearing number periphery	0.85			
Line crossings	0.81			0.41
Grooming number				0.69
Black and white box				
Rearing number white	0.40		0.73	
Grooming number		0.54	0.54	
Elevated plus maze				
Rearing number closed arm			0.83	
Percentage entries open arm	0.46			
Percentage time centre		−0.61		−0.59
Forced swim test				
Immobility 1st triad		0.89		
Struggling 2nd triad				0.81
Time swimming 2nd day		−0.88		
Percentage total variance	27.25	20.82	16.29	12.23

The most significant factor loadings, higher than 0.4, are shown for each behavioural parameter.

### Memory effects of Gsx

**Fig. 4:**
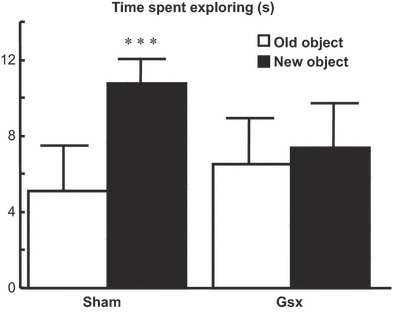
Time spent exploring during the recognition trial of the object recognition test. Rats from the sham group spent more time exploring the new object whereas rats from the Gsx gruop spent the same time exploring the old and new object. Sham, *n*=9; Gsx group, *n*=5. ***P<0.001.

One rat from each surgical group was excluded from the experimental protocol as they did not reach the cut-off of 20 s for the initial object exploration. During the recognition trial, rats from the sham group spent more time exploring the new object (*t*_12_ = 0.24, *P*=0.81, Fig. 4) whereas rats from the Gsx spent an equal amount of time exploring the old and new object (*t*_12_ = 4.47, *P*=0.0004, Fig. 4). Consequently, the percentage of time spent exploring the new object was higher in the sham group (*t*_12_ = 2.26, *P*=0.043).

### Content of monoamine and metabolites

In the prefrontal cortex there was no significant difference in the monoamines and metabolites measured between the sham-operated and Gsx rats (Table 5). In the nucleus accumbens, both the turnover of serotonin (5-HIAA/5-HT, *t*_11_ = 2.26, *P*=0.044) as well as dopamine (DOPAC/DA, *U*=6, *P*=0.02, *U*-test) were decreased in the Gsx rats (Table 5).

**Table 5:** Comparison of cerebral serotonin (5-HT), 5-hydroxiindolacetic acid (5-HIAA), turnover of serotonin (TU 5-HT), dopamine, (DA), dihydroxyphenylacetic acid (DOPAC), homovanillic acid (HVA) and turnover of dopamine (DOPAC/DA and HVA/DA) expressed in fmol/g of tissue between sham-operated (*n*=10) and Gsx rats (*n*=6)

	Prefrontal cortex		Nucleus accumbens	
		
	Sham	Gsx	Sham	Gsx
5-HT	619.83 ± 29.28	686.26 ± 44.97	946.35 ± 36.69	948.06 ± 33.25
5-HIAA	229.29 ± 12.19	211.80 ± 7.94	314.22 ± 14.01	281.02 ± 15.04
5-HIAA/5HT	0.37 ± 0.02	0.312 ± 0.02	0.33 ± 0.01	0.29 ± 0.01*
DA	402.73 ± 63.23	755.10 ± 313.03	3730.15 ± 160.49	3645.32 ± 551.23
DOPAC	357.18 ± 18.25	450.16 ± 71.89	913.07 ± 45.19	618.88 ± 167.26
HVA	47.52 ± 4.25	50.04 ± 14.31	155.45 ± 13.93	125.64 ± 18.81
DOPAC/DA	1.08 ± 0.21	0.91 ± 0.20	0.24 ± 0.01	0.15 ± 0.03*
HVA/DA	0.12 ± 0.02	0.08 ± 0.01	0.04 ± 0.00	0.04 ± 0.00

Values are expressed as mean ± SEM. *P < 0.05.

### Relative mRNA expression changes in amygdala and hippocampus

**Fig. 5:**
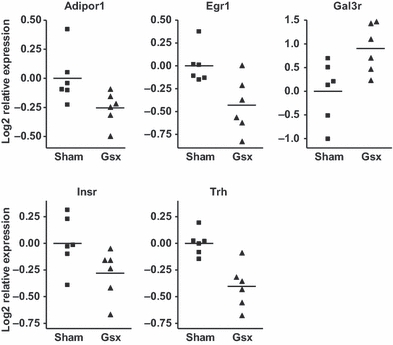
Effects of Gsx on hippocampal mRNA expression of adipor1 (adiponectin receptor 1), insulin receptor (insr), Egr-1 (early growth response protein 1), Trh (thyrotropin-releasing hormone) and Galr3 (galanin receptor 3) in comparison with sham-operation. *P*<0.05 for all comparisons.

To study the effect of Gsx on gene expression in brain structures involved in emotion and cognition (hippocampus and amygdala) we studied mRNA expression of genes implicated in these processes. A full list of the genes studied, including nomenclature/abbreviations, is given in Table 1. In the hippocampus the following genes were down-regulated in the Gsx rats when compared with the sham group: Adipor1 (adiponectin receptor 1, RQ = 0.84, *P =*0.017), Insr (insulin receptor, RQ = 0.82, *P =*0.013), Egr-1 (early growth response 1, RQ = 0.74, *P =*0.015) and Trh (thyrotropin releasing hormone, RQ = 0.76, *P =*0.021; Fig. 5A–D). The galanin receptor 3 (Gal 3) was upregulated (RQ = 1.87, *P =*0.023) in the hippocampus of Gsx rats (Fig. 5E). In the amygdala one gene was downregulated, namely Egr-1 (RQ = 0.89, *P =*0.035). None of the genes studied in neural growth/differentiation were different between the groups, namely BDNF (brain-derived neurotrophic factor), Ntrk2 (neurotrophic tyrosine kinase receptor 2), Ncam1 (neural cell adhesion molecule 1) and Ngfb (nerve growth factor beta) in any of the two structures (data not shown). The genes selected to investigate changes in the serotonin system (Htr1a, Htr2c and Sl6a4), the dopaminergic system (Ddr3, Th and Sl6a3) and the GABAergic system (Gabar1, Gabar3, Gabar4, Gabar5 and Gad1) were not changed after Gsx in either of the brain structures studied (data not shown).

### Hippocampal neurogenesis

The number of surviving neural precursor cells in the dentate gyrus was not significantly different between sham-operated and Gsx rats (*t*_12_ = 0.24, *P*=0.81) as detected by BrdU immunohistochemistry. The ratio of neuronal precursor cells maturing into neurons, reflected by ratio of the amount of NeuN/BrdU-positive double-labelled cells, was decreased in Gsx rats compared with sham-operated rats (70.0 vs. 82.5%, respectively, *t*_12_ = 2.84, *P*=0.015). However, when this ratio was multiplied by the total number of BrdU-positive cells acquired by stereology, this difference was lost (*t*_12_ = 1.02, *P*=0.326).

## Discussion

### Gastrectomy alters emotional reactivity in rats

Inspired by the clinical observation that Gsx patients have an altered emotional function and predisposition to depression/anxiety disorders, we demonstrate that Gsx rats differ from sham-operated rats in their emotional reactivity using tests that assess anxiety- and depression-like behaviour in rodents. To facilitate the interpretation of the large body of data (variables) emerging from these tests we used a powerful principal components (multivariate) analysis that enabled the identification of four ‘factors’ that account for most (around 77%) of the variance in emotional reactivity between the Gsx- and sham-operated rats. These factors could be considered to represent different traits of the emotional response that relate to anxiety- and depression-like behaviour. The final number of surviving operated rats in the two experimental groups could be considered to be relatively low for a behavioural study (i.e. six Gsx and 10 sham-operated) and with reduced statistical power it could therefore be difficult to show significant differences between the groups. However, it seems clear that the two experimental groups have a different emotional response.

From further consideration of the first emotionality factor/trait it is possible to deduce that spatial orientation and exploration of new surroundings are probably impaired in Gsx rats, as reflected in particular by the number of rearings in the peripheral area and the number of line crossings in the open field test. As rearing has been shown to involve central monoaminergic systems (Hofele *et al.*, 2001) we explored the turnover of dopamine (DOPAC/DA) and serotonin (5-HIAA/5-HT) in the nucleus accumbens and found that both were suppressed in Gsx rats. Given the role of the nucleus accumbens in the brain's reward system together with data describing the role of the stomach-derived hormone ghrelin in targeting the midbrain dopamine system (Jerlhag *et al.*, 2007) it is perhaps more likely that dopamine inputs from the ventral tegmental area as well as serotonergic projections from the dorsal raphe nucleus regulate accumbal monoamine turnover.

The most important variable within the second emotionality factor/trait of the multivariate analysis was time spent immobile in the forced swim test. Immobility is interpreted as an indicator of behavioural despair (Porsolt *et al.*, 1977; Cryan *et al.*, 2002) and in the Gsx rats time spent in this posture was decreased, indicating an anti-depressive effect of Gsx. This conclusion should, however, be made with some caution as we cannot rule out the possibility that differences in body weight between the two groups could have influenced the outcome of the forced swim test due to differences in buoyancy. In the open field and the black and white box there were trends in both tests for a decreased number of groomings in Gsx rats. As grooming behaviour and stress are closely linked this trend may be the effect of the reduced levels of corticosterone. Finally, the results of the forced swim test are not in accordance with the clinical situation in which ‘depression’ is described as part of an altered psychiatric profile in Gsx patients (Fujita *et al.*, 2003), for which there may be confounding factors and alternative explanations (e.g. post-cancer trauma and side-effects associated with malnutrition and dumping syndrome; Liedman, 1999).

The mechanism underpinning the effects of Gsx on emotional reactivity probably involves altered gut–brain endocrine signalling and also endocrine disturbances that are secondary to weight loss. Gsx is associated with suppressed levels of ghrelin, obestatin, gastrin, pancreastatin and leptin and with an increase in meal-associated CCK release (Inoue *et al.*, 1987; Dornonville de la Cour *et al.*, 2005; Furnes *et al.*, 2008b). Several of the hormones affected by Gsx have been shown previously to be associated with emotional responses when administered centrally. Intracerebroventricular injection of ghrelin has been shown to increase anxiety-like behaviour in a dose-dependent manner in rodents (Carlini *et al.*, 2002) whereas reduced central ghrelin signalling using antisense DNA for ghrelin causes an antidepressant and anxiolytic response in rats (Kanehisa *et al.*, 2006). Another study reported, surprisingly, beneficial effects of ghrelin to defend against depressive-like symptoms of chronic stress (Lutter *et al.*, 2008). Given that ghrelin levels are suppressed by Gsx, a chronic intervention, our recent study showing effects of chronic central ghrelin treatment increase anxiety-like behavior is especially relevant (Hansson *et al.*, 2011).

Increased anxiety-like behaviour has also been reported following central administration of obestatin (Carlini *et al.*, 2007). CCK is also considered anxiogenic and the effect of centrally administered CCK is mediated through the CCK-2 receptor (for a review see Wang *et al.*, 2005). Given that leptin is anxiolytic when administered centrally (Liu *et al.*, 2010), suppressed leptin in Gsx rats (Furnes *et al.*, 2008a) could also contribute to the altered emotional response. Of course, many of these hormones are also produced centrally, and so it remains of relevance whether they form an important afferent gut–brain signal for the control of mood in normal physiology. Our observation that there is a different emotional response between the two groups in the tests used to evaluate anxiety and depression further strengthens the evidence that gut peptides are involved in emotional processing. Although further experiments are required to demonstrate that these hormones mediate the altered emotionality following Gsx, the Gsx model provides insight regarding the possible consequences of depletion of these hormones on mood, thereby complementing studies of central hormone injection.

### Gastrectomy causes a memory deficit in rats

Gsx rats also spent less time exploring new objects in the object recognition test. This suggests a deficit in recognition memory, specifically working memory. However, the test is based on the spontaneous tendency of rats to explore a novel object over a familiar one and there are therefore alternative explanations that include decreased motivation to explore new objects or ‘novelty-seeking behaviour’. It is likely that the reduced gut peptide levels also cause this effect as gut peptides also play a role in memory function (Carlini *et al.*, 2008; Lo *et al.*, 2008) and in controlling hippocampal spine synapse density (Diano *et al.*, 2006). To study if the effects of Gsx on memory were caused by a decreased adult neurogenesis in the hippocampus, BrdU labelling as well as gene expression targeting nerve growth genes were performed. In the BrdU experiments no difference in the amount of proliferating cells or the number of neurons was observed between the surgical groups. In addition, we saw no changes in mRNA expression levels of the neural growth-associated genes studied, i.e. BDNF, NCAM 1, Ngfb or TrkB.

### Gastrectomy alters expression of genes implicated in emotional reactivity and memory/cognition in the hippocampus and amygdala of rats

Most changes in gene expression were observed in the hippocampus, in receptor genes associated with gut hormones or nutritional status but also involved in signal transduction. Hippocampal insulin receptor expression was down-regulated in Gsx rats. Insulin is important for cognition and hippocampal insulin receptor expression is increased in spatial memory training tasks (Zhao *et al.*, 1999) and insulin's enhancement of memory seems to be specific to the hippocampus (McNay, 2007). Insulin further influences central production of galanin (Tang *et al.*, 1997), a peptide described to have anxiolytic effects (Rajarao *et al.*, 2007). Interestingly, gene expression of the Galr2 receptor associated with the anti-depressant effects of galanin was unaltered in Gsx rats whereas Galr3 was distinctly up-regulated. The reduction in circulating ghrelin levels was not mirrored by a change in expression of its receptor (GHS-R1A), although it remains to be determined whether ghrelin effects on neuroplasticity are mediated via GHS-R1A only or whether they involve signalling via as yet unidentified receptors, including receptors for des-octanoyl ghrelin (Thompson *et al.*, 2004; Muccioli *et al.*, 2007). Ghrelin may also exert its effect through other routes, such as modulation of insulin sensitivity (Barazzoni *et al.*, 2008).

The Gsx rats also had a decreased expression of Egr-1 in both hippocampus and amygdala. Egr-1, also known as Krox-24, zif268, NGFI-A, ZENK and Tis8, encodes a zink finger transcription factor and plays a key role in cell proliferation and is the most widely studied in the fields of neural plasticity and cognition. Egr-1-deficient mice are unable to form long-term memories and have impaired long-term potentiation (Jones *et al.*, 2001) whereas cognitive enhancement is associated with an increase of Egr-1 expression in the hippocampus (Shirvalkar *et al.*, 2006). It should be noted that Egr-1 is up-regulated following chronic treatment with a variety of anti-depressant drugs (Bjartmar *et al.*, 2000). Although many neurotransmitter systems are known to induce the expression of Egr-1, the signalling pathways are not yet fully identified. Gene expression of Trh, an important neuropeptide associated with cognitive functions, was also decreased in the hippocampus of Gsx rats. Trh mimetics have been shown to improve memory function (Olson *et al.*, 1995) and interestingly pro-Trh mRNA levels are increased in the anterior hippocampus in rats trained in the Morris water maze paradigm (Aguilar-Valles *et al.*, 2007). Adiponectin, a hormone produced peripherally by adipocytes, is known to be increased by gastric surgery (Yang *et al.*, 2001). Adipor1 has been shown to act in the hypothalamus to activate elements of the insulin and leptin signalling pathways (Coope *et al.*, 2008). Adipor1 was down-regulated in the hippocampus of Gsx rats, possibly to compensate for the increased adiponectin levels. Even though there are no clear studies regarding adiponectin and cognition, leptin, also derived from fat tissue, has been shown to be involved in memory processing (Farr *et al.*, 2006). We can only speculate on the role of altered adiponectin levels due to weight loss, the hippocampal down-regulation of Adipor1 and importance for loss of memory function in Gsx rats.

## Conclusions

Gsx in rats leads to an altered emotional reactivity together with a memory deficit that is in line with the clinical observations in Gsx patients. These behaviour effects may be explained, at least in part, by reduced dopamine and serotonin turnover in the nucleus accumbens. The effects on cognition may be explained by changes in gene expression in receptors and peptides associated with memory function in the hippocampus. Taken together with the emerging neurobiology of the importance of gut–brain signalling in emotional and cognitive processes, it seems likely that the endocrine function of the stomach plays an important role in the altered emotional and cognitive responses.

## Abbreviations

5-HIAA: 5-hydroxyindoleacetic acid
5-HT: 5-hydroxytryptophan (serotonin)
Adipor1: adiponectin receptor 1
BDNF: brain-derived neurotrophic factor
BrdU: 5-bromo-2-deoxyuridine
DA: dopamine
DOPAC: 3,4-dihydroxyphenylacetic acid
Egr-1: early growth response protein 1
GABA1,3,4: gammabutyric acid receptors 1,3,4
GAD 1: glutamate decarboxylase 1
Gal3: galanin receptor 3
Gsx: gastrectomy
Htr1a: 2c, serotonin receptor 1a, 2c
HVA: 3-methoxy-4-hydroxyphenyl acetic acid
Insr: insulin receptor
Ncam1: neural cell adhesion molecule 1
NeuN: neuronal nuclei
Ngfb: nerve growth factor beta
Ntrk2: neurotrophic tyrosine kinase, receptor, type 2
Prox-1: prospero homeobox protein 1
Th: tyrosine hydroxylase
Trh: thyrotropin-releasing hormone
